# Supplementary material for: Uracil in the carbonaceous asteroid (162173) Ryugu
Source: Nat Commun. 2023 Mar 21;14:1292. doi: 10.1038/s41467-023-36904-3 (PMC10030641; doi:10.1038/s41467-023-36904-3)
Supplement: Supplementary file 1 — Supplementary Information [file 41467_2023_36904_MOESM1_ESM.pdf]

Supplementary Information for
“Uracil in the carbonaceous asteroid (162173) Ryugu”

Yasuhiro Oba, Toshiki Koga, Yoshinori Takano, Nanako O. Ogawa, Naohiko Ohkouchi, Kazunori Sasaki, Hajime Sato, Daniel P. Glavin, Jason P. Dworkin, Hiroshi Naraoka, Shogo Tachibana, Hisayoshi Yurimoto, Tomoki Nakamura, Takaaki Noguchi, Ryuji Okazaki, Hikaru Yabuta, Kanako Sakamoto, Toru Yada, Masahiro Nishimura, Aiko Nakato, Akiko Miyazaki, Kasumi Yogata, Masanao Abe, Tatsuaki Okada, Tomohiro Usui, Makoto Yoshikawa, Takanao Saiki, Satoshi Tanaka, Fuyuto Terui, Satoru Nakazawa, Sei-ichiro Watanabe, Yuichi Tsuda, Hayabusa2-initial-analysis SOM team

Contents

- List of Hayabusa2-initial-analysis SOM team members
- Supplementary Discussion
    - Bulk analysis of the samples A0106 and C0107
    - Importance of nitrogen (N) for intramolecular electron conjugation
    - Previous assessments for the possible contribution of terrestrial nucleobases
    - Search for purine nucleobases in the Ryugu and Orgueil extracts
    - A rough estimate for the relative abundance of the alkylated N-heterocyclic molecules depending on their carbon numbers
- Supplementary Figures 1–7
- Supplementary Table 1
- Supplementary References 1–30

The Hayabusa2-initial-analysis SOM team: Hiroshi Naraoka[1], Yoshinori Takano[2], Jason P. Dworkin[3], Kenji Hamase[4], Aogu Furusho[4], Minako Hashiguchi[5], Kazuhiko Fukushima[6], Dan Aoki[6], José C. Aponte[3], Eric T. Parker[3], Daniel P. Glavin[3], Hannah L. McLain[3,7,8], Jamie E. Elsila[3], Heather V. Graham[3], John M. Eiler[9], Philippe Schmitt-Kopplin[10,11,12], Norbert Hertkorn[10], Alexander Ruf[13,14,15], Francois-Regis Orthous-Daunay[16], Cédric Wolters[16], Junko Isa[17,18], Véronique Vuitton[16], Roland Thissen[19], Nanako O. Ogawa[2], Saburo Sakai[2], Toshihiro Yoshimura[2], Toshiki Koga[2], Haruna Sugahara[20], Naohiko Ohkouchi[2], Hajime Mita[21], Yoshihiro Furukawa[22], Yasuhiro Oba[23], Yoshito Chikaraishi[23], Takaaki Yoshikawa[24], Satoru Tanaka[25], Mayu Morita[25], Morihiko Onose[25], Daisuke Araoka[26], Fumie Kabashima[27], Kosuke Fujishima[17], Hajime Sato[28], Kazunori Sasaki[28,29], Kuniyuki Kano[30], Shin-ichiro M. Nomura[31], Junken Aoki[30], Tomoya Yamazaki[23], Yuki Kimura[23].

[1]Department of Earth and Planetary Sciences, Kyushu University, Fukuoka 819-0395, Japan.

[2]Biogeochemistry Research Center, Japan Agency for Marine-Earth Science and Technology, Yokosuka 237-0061, Japan.

[3]Solar System Exploration Division, NASA Goddard Space Flight Center, Greenbelt, MD 20771, USA.

[4]Graduate School of Pharmaceutical Sciences, Kyushu University, Fukuoka 812-8582, Japan.

[5]Graduate School of Environmental Studies, Nagoya University, Nagoya 464-8601, Japan.

[6]Graduate School of Bioagricultural Sciences, Nagoya University, Nagoya 464-8601, Japan.

[7]Center for Research and Exploration in Space Science and Technology, NASA Goddard Space Flight Center, Greenbelt, MD 20771, USA.

[8]Department of Physics, The Catholic University of America, Washington, DC 20064, USA.

[9]Division of Geological and Planetary Sciences, California Institute of Technology, Pasadena, CA 91125, USA.

[10]Helmholtz Munich, Analytical BioGeoChemistry, Neuherberg 85764, Germany.

[11]Technische Universität München, Analytische Lebensmittel Chemie, Freising 85354, Germany.

[12]Max Planck Institute for Extraterrestrial Physics, Garching bei München 85748, Germany.

[13]Université Aix-Marseille, CNRS, Laboratoire de Physique des Interactions Ioniques et Moléculaires, Marseille 13397, France.

[14]Department of Chemistry and Pharmacy, Ludwig-Maximilians-University, Munich 81377, Germany.

[15]Excellence Cluster ORIGINS, Garching 85748, Germany.

[16]Université Grenoble Alpes, Centre National de la Recherche Scientifique (CNRS), Centre National d'Etudes Spatiales, L'Institut de Planétologie et d'Astrophysique de Grenoble, Grenoble 38000, France.

[17]Earth-Life Science Institute, Tokyo Institute of Technology, Tokyo 152-8550, Japan.

[18]Planetary Exploration Research Center, Chiba Institute of Technology, Narashino 275-0016, Japan.

[19]Université Paris-Saclay, CNRS, Institut de Chimie Physique, Orsay 91405, France.

[20]Institute of Space and Astronautical Science, Japan Aerospace Exploration Agency (JAXA), Sagamihara 252-5210, Japan.

[21]Department of Life, Environment and Material Science, Fukuoka Institute of Technology, Fukuoka 811-0295, Japan.

[22]Department of Earth Science, Tohoku University, Sendai 980-8578, Japan.

[23]Institute of Low Temperature Science, Hokkaido University, Sapporo 060-0819, Japan.

[24]HORIBA Advanced Techno, Co., Ltd., Kyoto, 601-8305, Japan.

[25]HORIBA Techno Servoce Co., Ltd., Kyoto, 601-8305, Japan.

[26]National Institute of Advanced Industrial Science and Technology, Tsukuba, 305-8567, Japan

[27]LECO Japan Corp., Tokyo, 105-0014, Japan.

[28]Institute for Advanced Biosciences (IAB), Keio University, Kakuganji, Tsuruoka, Yamagata 997-0052, Japan.

[29]Human Metabolome Technologies Inc., Kakuganji, Tsuruoka, Yamagata 997-0052, Japan.

[30]Department of Health Chemistry, Graduate School of Pharmaceutical Sciences, The University of Tokyo Hongo, Tokyo 113-0033, JAPAN

[31]Department of Robotics, Graduate school of Engineering, Tohoku University, Sendai, Miyagi, 980-8579, JAPAN

Supplementary Discussion

Bulk analysis of the samples A0106 and C0107

Figure 1 shows photographs of initial samples for A0106 and C0107 collected from the 1st touchdown site and 2nd touchdown site, respectively[1,2]. Following up on the description of surface sample (A0106)[3], the subsurface Ryugu sample (C0107) contained 3.58 ± 0.47 wt% (n = 4; sample weight of each measurement = 126.3 ± 56.7 μg) of total carbon with the isotopic composition ($\delta^{13}C$ = +1.22 ± 10.0‰, n = 4), 1.06 ± 0.10 wt% (n = 3; average weight = 373.0 ± 137.5 μg) of total hydrogen (δD = +269 ± 13‰, n = 3), 0.14 ± 0.01 wt% (n = 4; sample weight = 126.3 ± 56.7 μg) of total nitrogen ($\delta^{15}N$ = +36.8 ± 3.6‰, n = 3), and 5.5 ± 0.7 wt% (n = 5; sample weight = 20.6 ± 5.4 μg) of total sulfur ($\delta^{34}S$ = -1.10 ± 1.62‰, n = 5). The concentration of pyrolyzed oxygen was 13.4 ± 1.0 wt% (n = 3; sample weight = 373.0 ± 137.5 μg) with the isotopic composition for $\delta^{18}O$ = +12.1 ± 1.2‰ (n = 3). Evaluating the elemental ratios, we find that the weight (wt%)-based elemental ratios converge as follows: C/N ratio = 26.0 ± 2.4, O/H ratio = 12.7 ± 0.4, C/S ratios = 0.66 for Ryugu C0107. The isotopic differences between A0106 and C0107 for CHNSO indicated that slight isotopic heterogeneity was found in the samples from the first and second touchdown sites. The assessment of sample heterogeneity must also take into account potential regional differences between two touchdown sites[2]. The data profiles shown in Figure 2 are compiled from the following references[3–19].

Importance of nitrogen (N) for intramolecular electron conjugation

Since the valence of atomic nitrogen (N) is three, the remaining electron always form the lone electron pair (≡N:) in N-heterocyclic molecules (see Supplementary Fig. 1). The electron conjugation systems in nitrogen-containing compounds have a versatile role in molecular evolution in that they also have a significant impact on secondary molecular formation, molecular diversity, and molecular yield[20]. The Supplementary Fig. 2 shows the "pyrimidine ($C_4H_4N_2$)" molecule of the uracil skeleton discussed in this report with the electrostatic density as ground state. It is emphasized here that a hexagonal reference of "cyclohexane (C_6H_{12})" consisting only of a carbon backbone, has no electrostatic electronic conjugation system. Therefore, intramolecular electron conjugated systems that encapsulate nitrogen atoms contribute to potential diverse reaction systems and have important molecular evolutionary implications[21,22].

Previous assessments for the possible contribution of terrestrial nucleobases

Stoks & Schwartz[23] concluded that the detected uracil was indigenous to meteorites

since the uracil-to-thymine ratio in meteorites was clearly different from that in biogenic products. Martins et al.[24] performed a compound-specific carbon isotopic analysis of nucleobases in the HCOOH extract from the Murchison meteorite. The reported $\delta^{13}C$ values were +44.5 and +37.7‰ for uracil and xanthine, respectively, implying their extraterrestrial origin. However, due to the possible contribution from other meteoritic organic molecules to the measured $\delta^{13}C$ values, it remains uncertain whether the reported $\delta^{13}C$ values are derived from nucleobases or not[25]. Callahan et al.[25] proposed the indigeneity of the detected nucleobases based on the detection of purine and 6,8-diaminopurine in the same meteorite, both of which are rare in terrestrial samples. In addition, structural diversity typically observed for organic compounds in meteorites might be suggestive of their extraterrestrial origin[25]. Based on those pioneering findings, Oba et al.[26] detected various nucleobases in meteorites including Murchison. Since the amino acids such as alanine detected in the same specimen of the Murchison were racemic, the indigenous amino acids should be abiotic in origin[27], which suggests that the nucleobases in the same specimen are also abiotic in origin. This is a valuable opportunity to make first-hand observations on the molecular evolution of prebiotic amino acids and nucleobases[28].

Search for purine nucleobases in the Ryugu and Orgueil extracts

We detected uracil in both the Ryugu and Orgueil samples using a Hypercarb™ separation column, which is suitable for the detection of pyrimidine nucleobases[26] (Figure 4). However, the detection of purine nucleobases is not the case probably due to the strong interaction between purine nucleobases and the stationary phase of the Hypercarb column[26]. Hence, any purine nucleobases were not detected in the initial analysis of the Ryugu samples at the *m/z* range of 50–600. Although purine nucleobases may be detected if we use a different analytical column (InertSustain PFP, GL Science)[26], due to the limitation on the sample volume, we could not search for these molecules using this column. On the other hand, purine nucleobases may be present in the Orgueil extract as has been reported in Callahan et al. [25] (20 ppb for guanine and 7 ppb for adenine). However, any purine nucleobases were not detected in the Orgueil extract after acid hydrolysis even when a PFP column was used. A rough estimate for the detection limit of purine nucleobases was 1 ppb under the present analytical conditions where ~10 mg of samples was used. Since the concentrations of purine nucleobases reported in Callahan et al. [25] were higher than the detection limit, they are likely to be detected in the present study. The non-detection in the present study may be attributable to the heterogeneous distribution in Orgueil meteorite and/or different extraction methods (hot water extraction in the present study vs. formic acid extraction in

Callahan et al.[25]).

A rough estimate for the relative abundance of the alkylated N-heterocyclic molecules depending on their carbon numbers

Fig. 7 and Supplementary Fig. 7 show variations in the sum of peak area at the *m/z* corresponding to alkylated homologues of N-heterocyclic molecules (Supplementary Fig. 6). Under the present analytical precision (within 3 ppm), a monoisotopic mass (e.g. 113.0346) is linked to a single molecular formulae ($C_4H_4N_2O_2$+H^+). Assuming that the ionization efficiency is unity among the same classes of molecules, the variations in the sum of peak area are equivalent to those in the concentration of molecules. Note that the peak area of a certain compound (e.g., alkylimidazole) cannot be compared with others (e.g. alkylpyridine) since their ionization efficiencies would not be unity. In addition, some of N-heterocyclic molecules may have their structural isomers (e.g., uracil and imidazole carboxylic acids), which cannot be distinguished on the mass chromatograms due to the low peak resolutions. In that case, the sum of peak area represents the sum of these structural isomers.

Even under those situations, they can be compared with those in other samples, which may be helpful for discussing the formation/alteration histories on the parent body. In principle, the variations in the peak are for N-heterocyclic molecules are similar between A0106 and C0107 (Fig. 7, Supplementary Fig. 7). However, small but not negligible discrepancy is observed between the two samples. Although both samples are similar in that each N-heterocycle shows unimodal or bimodal distribution with relevance to their carbon numbers, there is a slight difference in the distribution. For example, alkyloxazole shows the highest peak at the carbon number of C_{15} for A0106 but at C_{13} for C0107 (Supplementary Fig. 7). As a general trend, the molecular size seems to be larger for A0107 than C0107. Assuming that A0106, which is surface materials, experiences more extensive alteration processes than C0107 by external energies (Fig. 8), the larger size of N-heterocyclic molecules may be the result of such processes. UV and cosmic ray often cause bond dissociation of molecules to yield smaller reactive radicals. If such radicals are present nearby, they could recombine to form larger species. In contrast, such processes would simply result in the dissociation of N-heterocyclic molecules. In case N atoms are released upon degradation, it is anticipated that PAHs are formed accordingly. This story is consistent with the larger abundances of PAHs in the A0106 than in the C0107[29], but further investigation must be necessary to conclude.

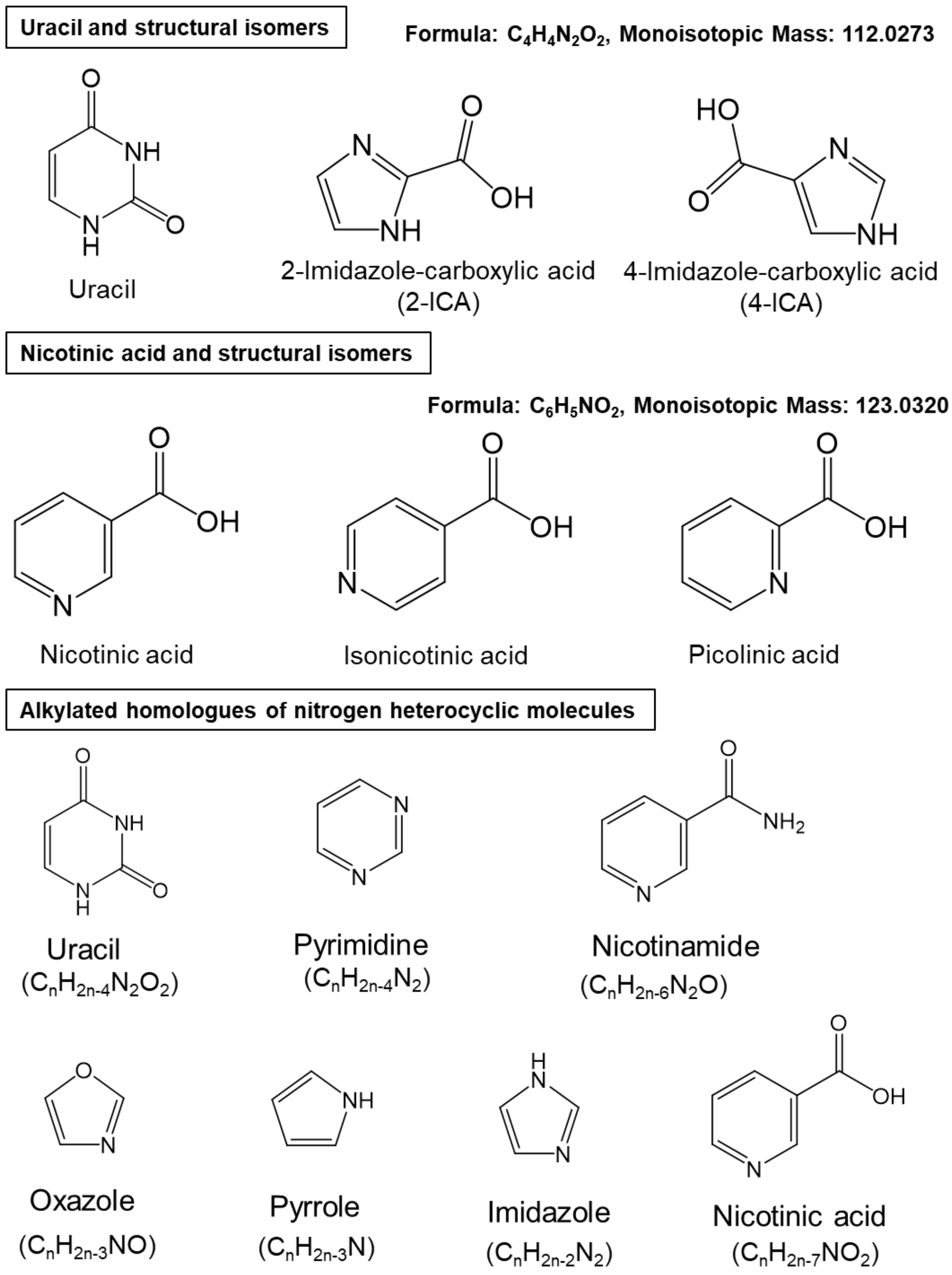

Supplementary Figure 1. Molecular structures of N-heterocyclic molecules targeted in the present study. Uracil and its structural isomers, 2-imidazole-carboxylic acid and 4-imidazole-carboxylic acid, nicotinic acid and its structural isomer isonicotinic acid were detected in the acid-hydrolysate of the hot water extracts from the Ryugu samples. In the methanol extract, alkylated homologues of uracil ($C_nH_{2n-4}N_2O_2$), pyrimidine ($C_nH_{2n-4}N_2$), nicotinamide ($C_nH_{2n-6}N_2O$), oxazole ($C_nH_{2n-3}NO$), pyrrole ($C_nH_{2n-3}N_2$), imidazole ($C_nH_{2n-2}N_2$), and nicotinic acid ($C_nH_{2n-7}NO_2$) were expected to be present where *n* represents the number of carbon atoms in a molecule.

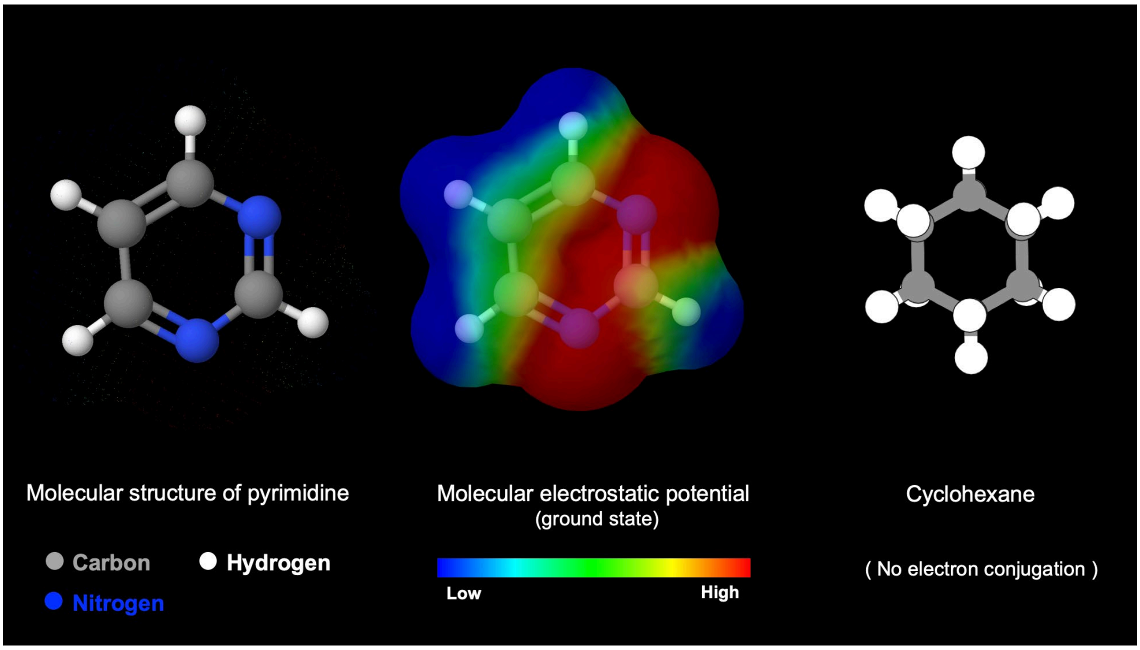

Supplementary Figure 2. Molecular structure and electrostatic potential in the ground state of pyrimidine ($C_4H_4N_2$) and cyclohaxane (C_6H_{12}). Due to its variations in the electron densities, N-heterocyclic molecules generally possess higher reactivity than corresponding cyclic hydrocarbons which do not have any electron conjugation in its structure.

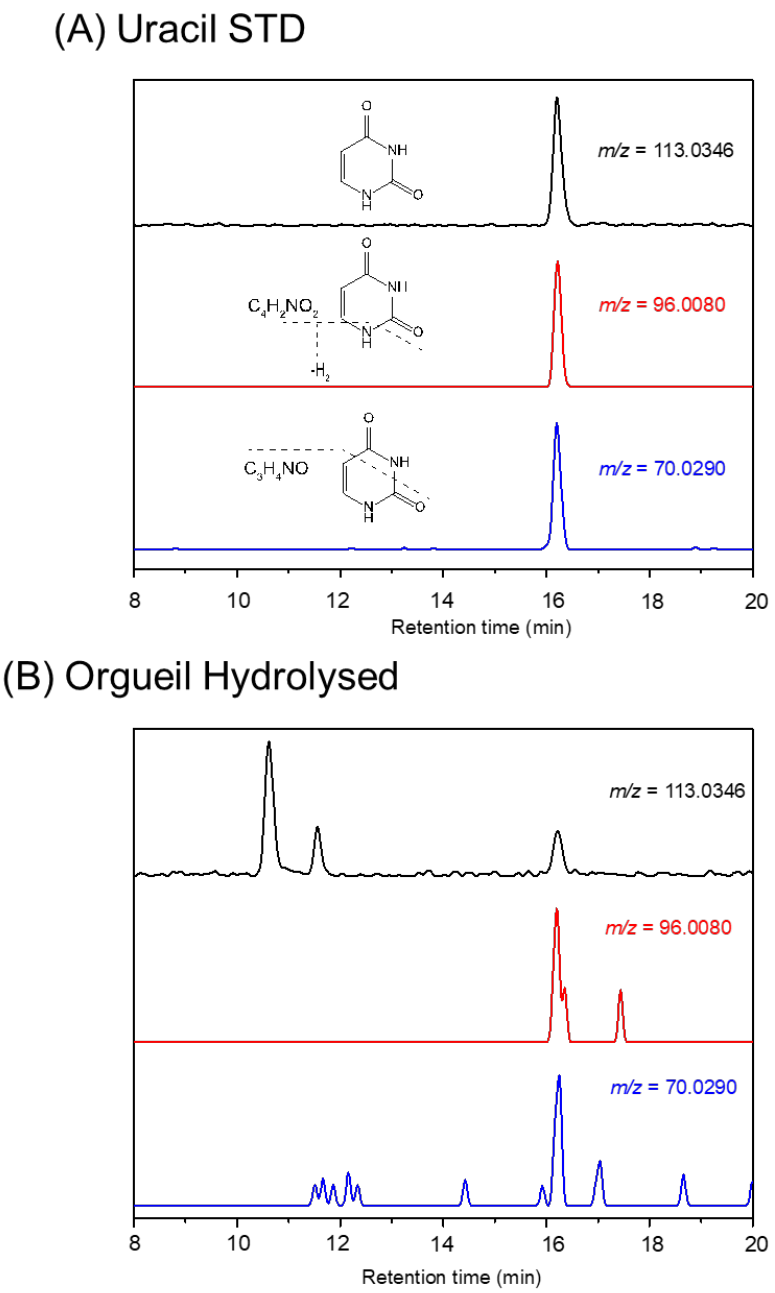

Supplementary Figure 3. MS/MS measurements of uracil standard reagents and the acid hydrolysate of the Orgueil extract. Extracted mass chromatograms of (A) the uracil standard reagent and (B) the acid hydrolysate of Orgueil extract in the MS/MS measurements. The *m/z* of 113.0346 represents the parent ion, and that of 96.0080 and 70.0290 represents its fragment ions. The fragmentation patterns are referred to Oba et al.[26]. The observed mass fragmentation patters at 16 min in the Orgueil CI meteorite are well consistent with those of the authentic standard of uracil.

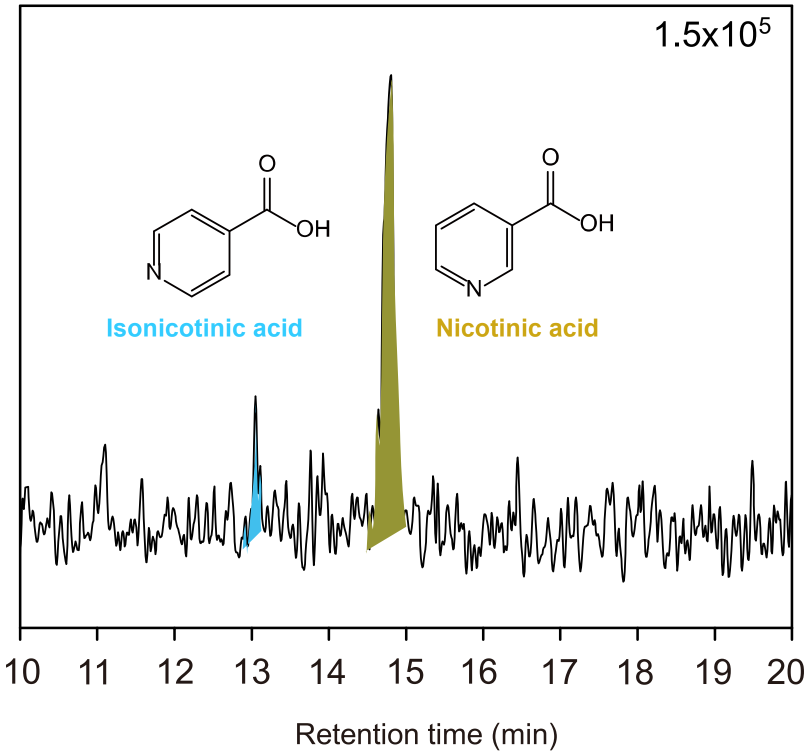

Supplementary Figure 4. Nicotinic acid and isonicotinic acid in organic residues synthesized in laboratory. Both nicotinic and isonicotinic acids were identified in organic residues produced by the photolysis of interstellar ice analogues composed of H_2O, CO, CH_3OH, and NH_3 at 10 K[26,30]. Analytical conditions were different from those applied in the present study. See Oba et al[30]. Picolinic acid was not positively identified. The nicotinic acid/isonicotinic acid ratio was ~3.2 in this product. The number on the top right represents the relative intensity at the chromatogram.

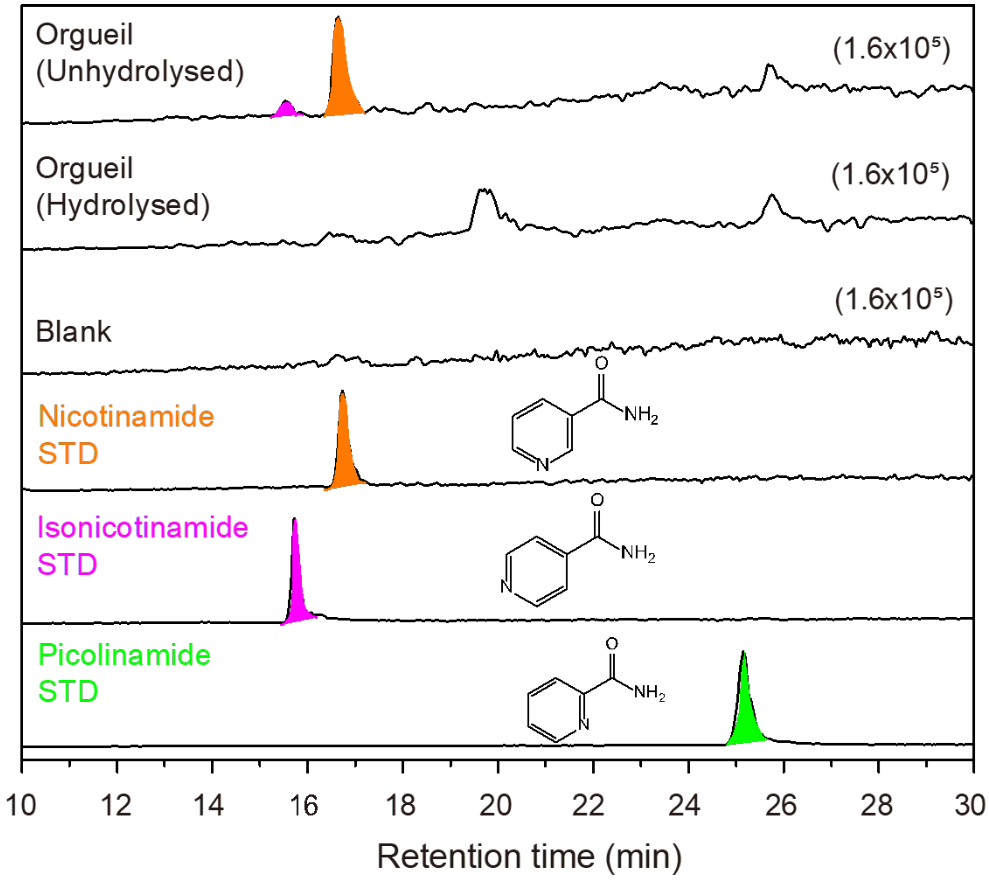

Supplementary Figure 5. Nicotinamide and isonicotinamide detected in the Orgueil extract. LC/HRMS chromatograms at the *m/z* of 123.0553 which corresponds to the exact mass number for the protonated ion of nicotinamide and its structural isomers in the hot water extract and its acid hydrolysate from the Orgueil meteorite. Data for the blank sample and authentic standards are also shown for comparison. Numbers in parenthesis represents the relative intensity at each chromatogram. The observed peak at ~26 min on the mass chromatograms for the Orgueil extract was confirmed to be different from picolinamide by MS/MS measurements.

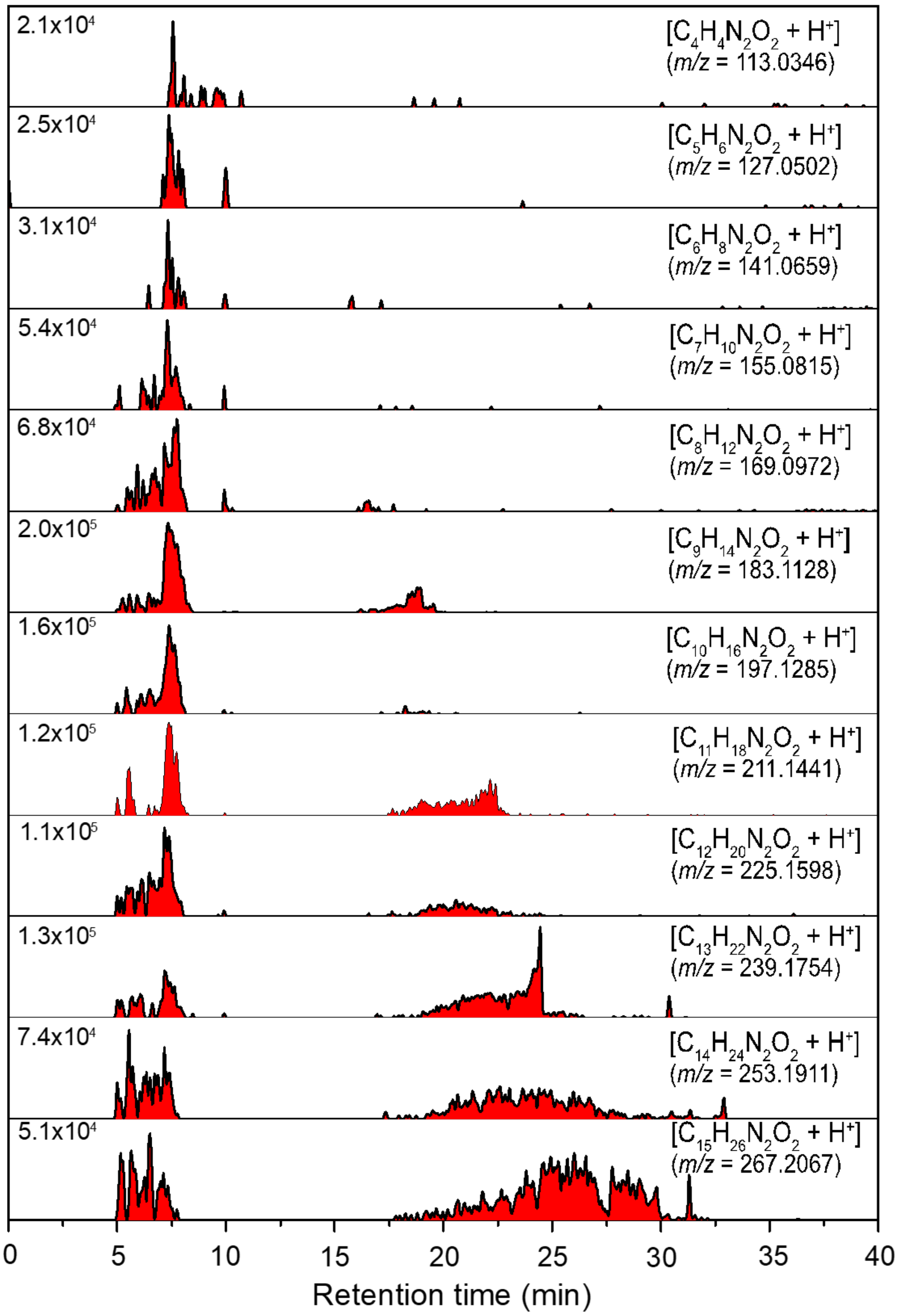

Supplementary Figure 6. Detection of $C_nH_{2n-4}N_2O_2$ molecules from the extract of A0106. Mass chromatograms at the *m/z* corresponding to $C_nH_{2n-4}N_2O_2$ molecules with the carbon number (*n*) of 4 to 15. Alkylated analogues of uracil and imidazole-carboxylic acids are represented by this molecular formula. Numbers on the left indicate the relative intensities for each chromatogram.

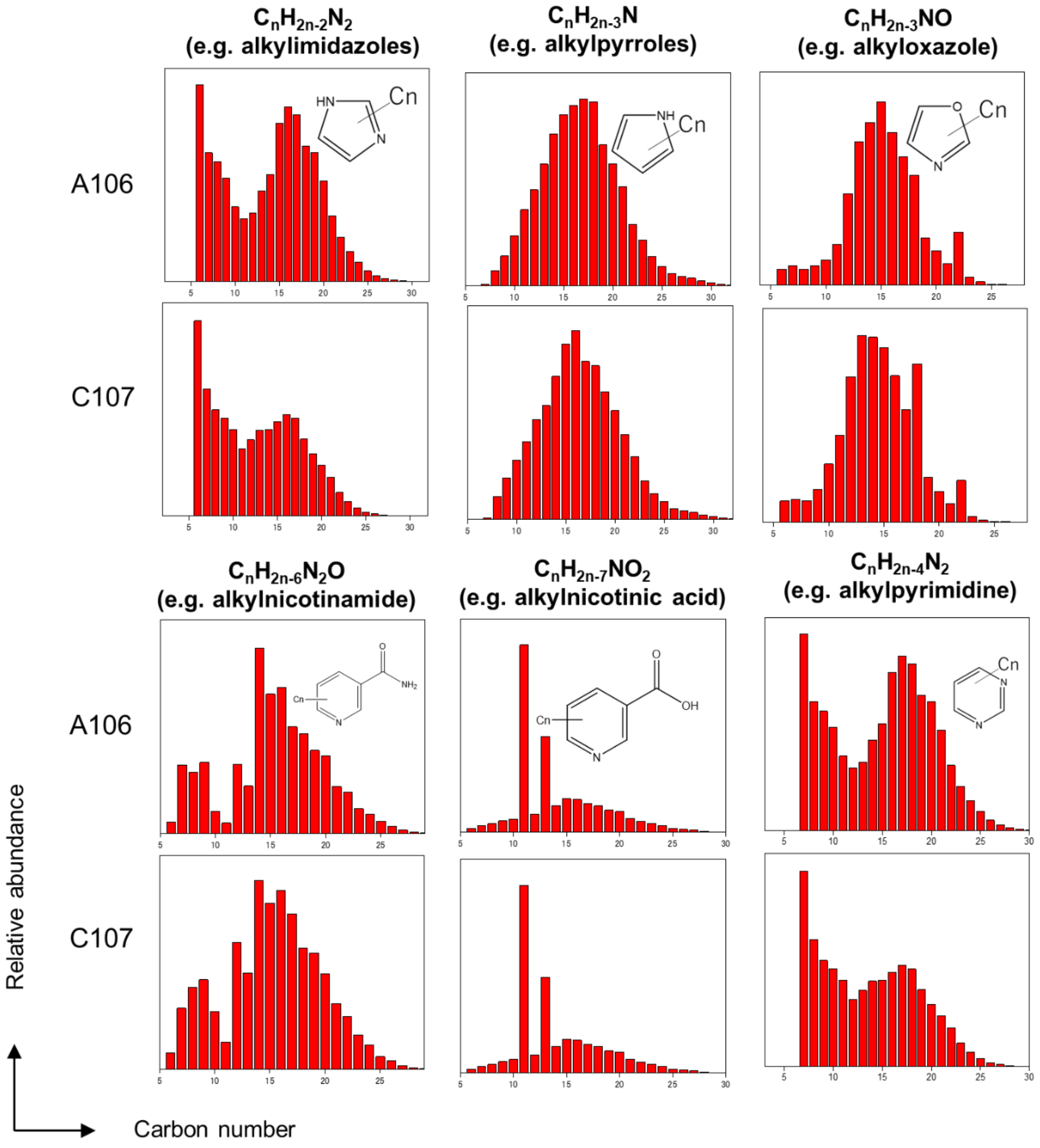

Supplementary Figure 7. Relative abundance of alkylated CHNO molecules in the A0106 and C0107 samples. Comparison of the sum of peak area at the *m/z* ratio corresponding to the alkylated homologues of CHNO molecules having a general formula $C_nH_{2n-2}N_2$, $C_nH_{2n-3}N$, $C_nH_{2n-3}NO$, $C_nH_{2n-6}N_2O$, $C_nH_{2n-7}NO_2$, and $C_nH_{2n-4}N_2$ (n = 5 to 30), which include imidazole, pyrrole, oxazole, nicotinamide, nicotinic acid, and pyrimidine, respectively, between A0106 and C0107 samples. The horizontal axis shows the number of carbons (C*n*-) and the vertical axis shows the sum of peak area at each *m/z*.

Supplementary Table 1. Raw data for the elemental and isotopic compositions of Ryugu grains C0107 shown in Figure 2.

Ryugu C0107	Shape	Weight (ug)	1σ	Carbon (wt%)	1σ	δ^{13}C (‰ vs. VPDB)	1σ	Nitrogen (wt%)	1σ	δ^{15}N (‰ vs. Air)	1σ	weight C/N ratio	1σ
C0107_Initial Bulk_#1	small grains	45.0		3.36		-7.4		0.12				27.0	
C0107_Initial Bulk_#2	small grains	130.5		3.24		-1.3		0.13		39.0		24.0	
C0107_Initial Bulk_#3	small grains	160.0		3.47		-2.1		0.14		32.6		24.1	
C0107_Initial Bulk_#4	small grains	169.6		4.27		15.7		0.15		38.8		28.9	
**Average (*n* = 4)**		**126.3**	**±56.7**	**3.58**	**±0.47**	**1.22**	**±10.0**	**0.14**	**±0.01**	**36.8**	**±3.6**	**26.0**	**±2.4**
	Shape	Weight (ug)	1σ	Hydrogen (wt%)	1σ	δD (‰ vs. VSMOW)	1σ	Oxygen (wt%)*	1σ	δ^{18}O (‰ vs. VSMOW	1σ	weight O/H ratio	1σ
C0107_Initial Bulk_#5	small grains	527.6		1.12		255		13.8		11.0		12.3	
C0107_Initial Bulk_#6	small grains	327.2		1.10		281		14.1		12.0		12.8	
C0107_Initial Bulk_#7	small grains	264.2		0.94		270		12.3		13.4		13.0	
**Average (*n* = 3)**		**373.0**	**±137.5**	**1.06**	**±0.10**	**269**	**±13**	**13.4**	**±1.0**	**12.1**	**±1.2**	**12.7**	**±0.4**
	Shape	Weight (ug)	1σ	Sulfur (wt%)	1σ	δ^{34}S (‰ vs. VCDT)	1σ					weight C/S ratio	
C0107_Initial Bulk_#8	small grains	29.8		5.77		-2.5						-	
C0107_Initial Bulk_#9	small grains	18.5		5.25		1.7						-	
C0107_Initial Bulk_#10	small grains	16.2		5.07		-1.5						-	
C0107_Initial Bulk_#11	small grains	17.8		4.76		-2.0						-	
C0107_Initial Bulk_#12	small grains	20.6		6.49		-1.2						-	
**Average (*n* = 5)**		**20.6**	**±5.4**	**5.47**	**±0.7**	**-1.10**	**±1.62**				Ave./Ave	**0.66**	

*The concentration of pyrolyzed oxygen (i.e., thermally released oxygen, except for anhydrous silicate minerals). The isotope ratios of light elements are shown in each international standard scale.

Note: The raw data for A0106 have been shown in ref. 3.

Supplementary References

1. Yada, T. et al. Preliminary analysis of the Hayabusa2 samples returned from C-type asteroid Ryugu. *Nature Astron.* **6**, 214–220 (2022).
2. Tachibana, S. et al. Pebbles and sans on asteroid (162173) Ryugu: On-site observation and returned particles from two landing sites. *Science* **375**, 1011-1016 (2022).
3. Naraoka, H. et al. Soluble organic molecules in samples of the carbonaceous asteroid (162173) Ryugu. *Science,* doi: 10.1126/science.abn9033 (2023)
4. Boato, G. The isotopic composition of hydrogen and carbon in the carbonaceous chondrites. *Geochim. Cosmochim. Acta* **6**, 209–220 (1954).
5. Robert, F. et al. Deuterium concentration in the early Solar System: Hydrogen and oxygen isotope study. *Nature* **282**, 785–789 (1979).
6. Kolodny, Y. et al. Deuterium in carbonaceous chondrites. *Earth Planet. Sci. Lett.* **46**, 149–158 (1980).
7. McNaughton, N. et al. Deuterium/hydrogen ratios in unequilibrated ordinary chondrites. *Nature* **294**, 639–641 (1981).
8. Robert, F., Epstein, S. The concentration and isotopic composition of hydrogen, carbon and nitrogen in carbonaceous meteorites. *Geochim. Cosmochim. Acta* **46**, 81–95 (1982).
9. Yang, J., Epstein, S. Interstellar organic matter in meteorites. *Geochim. Cosmochim. Acta* **47**, 2199–2216 (1983).
10. Kerridge, J. F. Carbon, hydrogen and nitrogen in carbonaceous chondrites: Abundances and isotopic compositions in bulk samples. *Geochim. Cosmochim. Acta* **49**, 1707–1714 (1985).
11. Pearson, V. K., Sephton, M. and Gilmour, I. Molecular and isotopic indicators of alteration in CR chondrites. *Meteorit. Planet. Sci.* **41**, 1291–1303 (2006).
12. Jarosewich, E. Chemical analyses of meteorites at the Smithsonian Institution: An update. *Meteorit. Planet. Sci.* **41**, 1381–1382 (2006).
13. Alexander C. et al., The Provenances of Asteroids, and Their Contributions to the Volatile Inventories of the Terrestrial Planets. *Science* **337**, 721–723 (2012).
14. Alexander C. et al., A multi-technique search for the most primitive CO chondrites. *Geochim. Cosmochim. Acta* **221**, 406–420 (2018).
15. Vacher, L. G. et al. Inward radial mixing of interstellar water ices in the solar protoplanetary disk. *Astrophys. J.* **827**, L1 (2016).
16. Vacher, L. G. et al. Hydrogen in chondrites: Influence of parent body alteration and atmospheric contamination on primordial components. *Geochim. Cosmochim. Acta* **281**, 53–

66 (2020).

17. Piani, L. et al. A dual origin for water in carbonaceous asteroids revealed by CM chondrites. *Nature Astron.* **2**, 317–323 (2018).
18. Piani, L. et al. Earth's water may have been inherited from material similar to enstatite chondrite meteorites. *Science* **369**, 1110–1113 (2020).
19. Piani, L. et al. Origin of hydrogen isotopic variations in chondritic water and organics. *Earth Planet. Sci. Lett.* **567**, 117008 (2021).
20. Rodriguez, L.E., House, C.H., Smith, K.E., Roberts, M.R. and Callahan, M.P. Nitrogen heterocycles form peptide nucleic acid precursors in complex prebiotic mixtures. *Sci. Rep.* **9**, Article number: 9281 (2019).
21. Ferris, J.P. and Hagan Jr, W.J. HCN and chemical evolution: the possible role of cyano compounds in prebiotic synthesis. *Tetrahedron* **40**, 1093-1120 (1984).
22. Cronin, J.R. and Chang, S. Organic matter in meteorites: Molecular and isotopic analyses of the Murchison meteorite, *The chemistry of life's origins.* Springer, pp. 209-258 (1993).
23. Stoks, P.G. and Schwartz, A.W. Uracil in carbonaceous meteorites. *Nature* **282**, 709-710 (1979).
24. Martins, Z. et al. Extraterrestrial nucleobases in the Murchison meteorite. *Earth. Planet. Sci. Lett.* **270**, 130–136 (2008).
25. Callahan, M.P. et al. Carbonaceous meteorites contain a wide range of extraterrestrial nucleobases. *PNAS* **108**, 13995-13998 (2011).
26. Oba, Y. et al. Identifying the wide diversity of extraterrestrial purine and pyrimidine nucleobases in carbonaceous meteorites. *Nature Commun.* **13**, Article number: 2008 (2022).
27. Friedrich, J.M. et al. Effect of polychromatic X-ray microtomography imaging on the amino acid content of the Murchison CM chondrite. *Meteor. Planet. Sci.* **54**, 220-228 (2019).
28. Burton, A. et al. Understanding prebiotic chemistry through the analysis of extraterrestrial amino acids and nucleobases in meteorites. *Chem. Soc. Rev.* **41**, 5459 – 5472 (2012).
29. Aponte, J. et al. Organic-soluble compounds in asteroid Ryugu samples A0106 and C0107 and the Orgueil (CI1) meteorite. *Earth Planet Space*, in press. (2023).
30. Oba, Y. et al. Nucleobase synthesis in interstellar ices. *Nature Commun.* **10**, Article number: 4431 (2019).
